# A Comprehensive Analysis of the Current Status and Unmet Needs in Kidney Transplantation in Southeast Asia

**DOI:** 10.3389/fmed.2017.00084

**Published:** 2017-06-23

**Authors:** Chitranon Chan-on, Minnie M. Sarwal

**Affiliations:** ^1^Division of Nephrology, Faculty of Medicine, Department of Internal Medicine, Khon Kaen University, Khon Kaen, Thailand; ^2^Division of Transplant Surgery, Department of Surgery, University of California, San Francisco, San Francisco, CA, United States

**Keywords:** organ donation, organ access, transplant policy, transplant law, kidney transplantation, health financing, donor compensation, kidney graft survival

## Abstract

To address the unmet needs in the face of a growing demand for end-stage renal failure management and kidney transplantation in Asia, we have conducted a critical analysis of published literature and national registries to evaluate clinical outcomes and the rates of organ donation in Southeast Asia and the challenges facing these regions with regards to regulation, choice of donor source, and funding. Based on the available data, suggestions are proposed for an advancement of rates of organ donation and access, with emphasis on improved regulation and public education.

## Introduction

Organ transplantation (tx) is the treatment of choice for patients who suffer from end organ failure (1–3). With the increasing morbidity of organ failure due to the evolution of dietary and environmental triggers, there is increasing pressure on the tx wait-lists that do not match the growth in available transplants. Improved health care accessibility and longevity also result in a growing population of individuals with end-stage renal disease (ESRD) (4, 5). Global tx activity in 2014 rose by 1.81% from 2013, which only meet ~ 10% of the global need for new organs (6). Asia is the most populated region in the world but, conversely, has the lowest rate of organ tx and the greatest growth rate for numbers of people entering chronic and end-stage organ failure (6). In 2014, in Southeast Asia, the rate of organ tx was 3.8 patients per million population (ppmp), compared to 31.6 ppmp in the US and 27.9 ppmp in Europe (6). This discrepancy in morbidity and organ availability and vast social, economic, and political diversity in the region has fostered organ trafficking in Southeast Asia (7). The inequities in health care accessibility are further complicated in this region by epidemiological pressures (8), policies on health care financing (9), and medical technology diffusion (10). To update the impact of these changes on tx provisions in Southeast Asia, we reviewed the tx rate, the burden of organ shortage, organ access, legislation framework, reimbursement, and clinical outcome data in this continent. We also examined the causes of the unmet needs for organ tx in this region, compared to similar data from the US as an international benchmark, with a view to understanding regional pressures that can help to improve access and outcomes of organ transplantation in this region.

We searched the PUBMED electronic database for English-language peer-reviewed reports, computerized databases, government reports, dissertations, and online national registries in each of the countries in Southeast Asia. Relevant articles between 1956 and 2016 were reviewed for additional information as the databases and registries may not provide complete data due to the voluntary nature of their reporting.

The Southeast Asian continent has several strata of countries with wide variations in the gross national income per capita statistics from the World Bank (11) and human development index (HDI) from United Nations Development Program (12, 13). HDI is a composite index of the achievement in longevity, education, and income, which are three key dimensions of human development (13–15). There is evidence for an association of organ tx access with national income and HDI (10). Thus, we also provide income and HDI at a glance before exploring tx services in different countries in this region.

**Figure 1:**
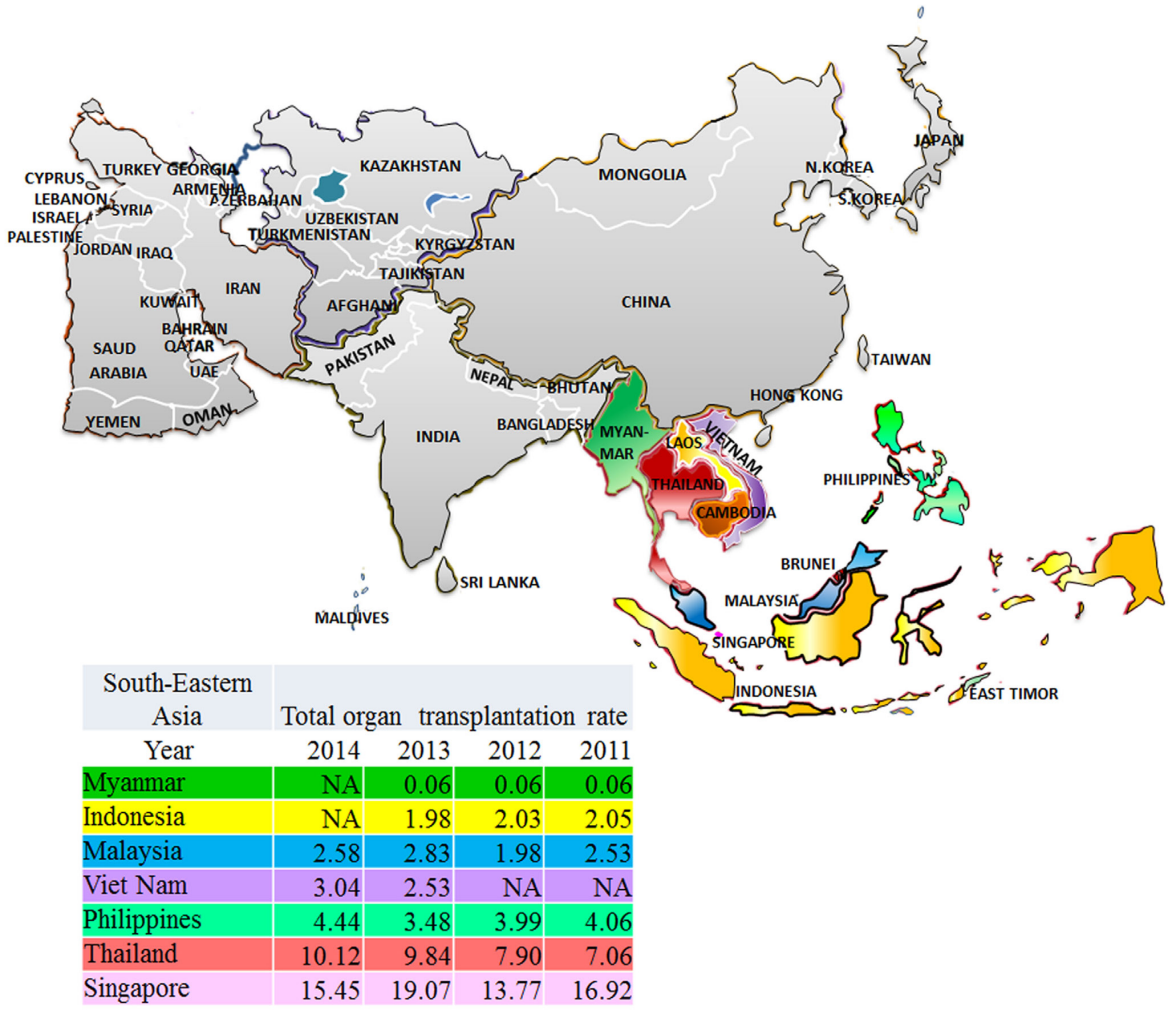
2011–2014 organ transplantation rates in Southeast Asia, patients per million population.

**Figure 2:**
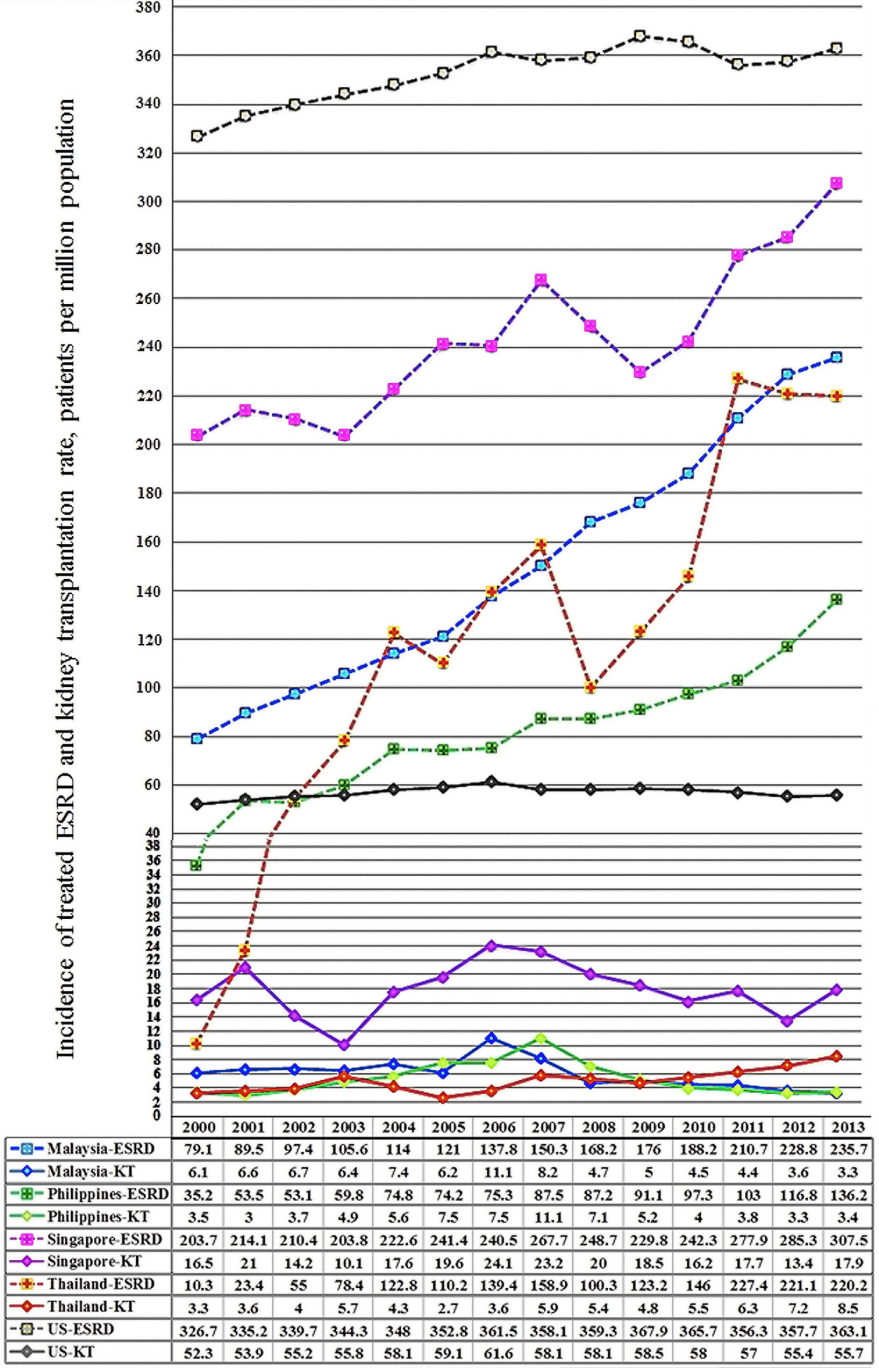
2000–2013 incidence of treated end-stage renal disease (ESRD) and kidney transplantation rates in Southeast Asia and the US, patients per million population.

The Southeast Asian region has been defined following the division of geographic areas as assessed by the United Nations Statistic Division revised September 26th, 2016 (16). Southeast Asia consists of 11 members (Figure 1) ranked from high-income economies like Singapore and Brunei Darussalam to low-income economies like Cambodia, Laos, Myanmar, and Timor-Leste. Singapore is in the very high HDI while Myanmar is in the lowest. The trend of increased incidence of treated ESRD in developing countries is marked in this region (Figure 2). From the 2015 United States Renal Data System Annual data report, the greatest proportional increases in the incidence of treated ESRD over the interval from 2000/1 to 2012/13 were seen in the following countries (8): Thailand (1,210%), Philippines (185%), and Malaysia (176%). The reasons are because of improved standards of clinical care (17, 18), increased access to medical treatment (9), and longer life expectancy (19).

## Singapore

### Organ Access, Legislation, and Reimbursement

**Figure 3:**
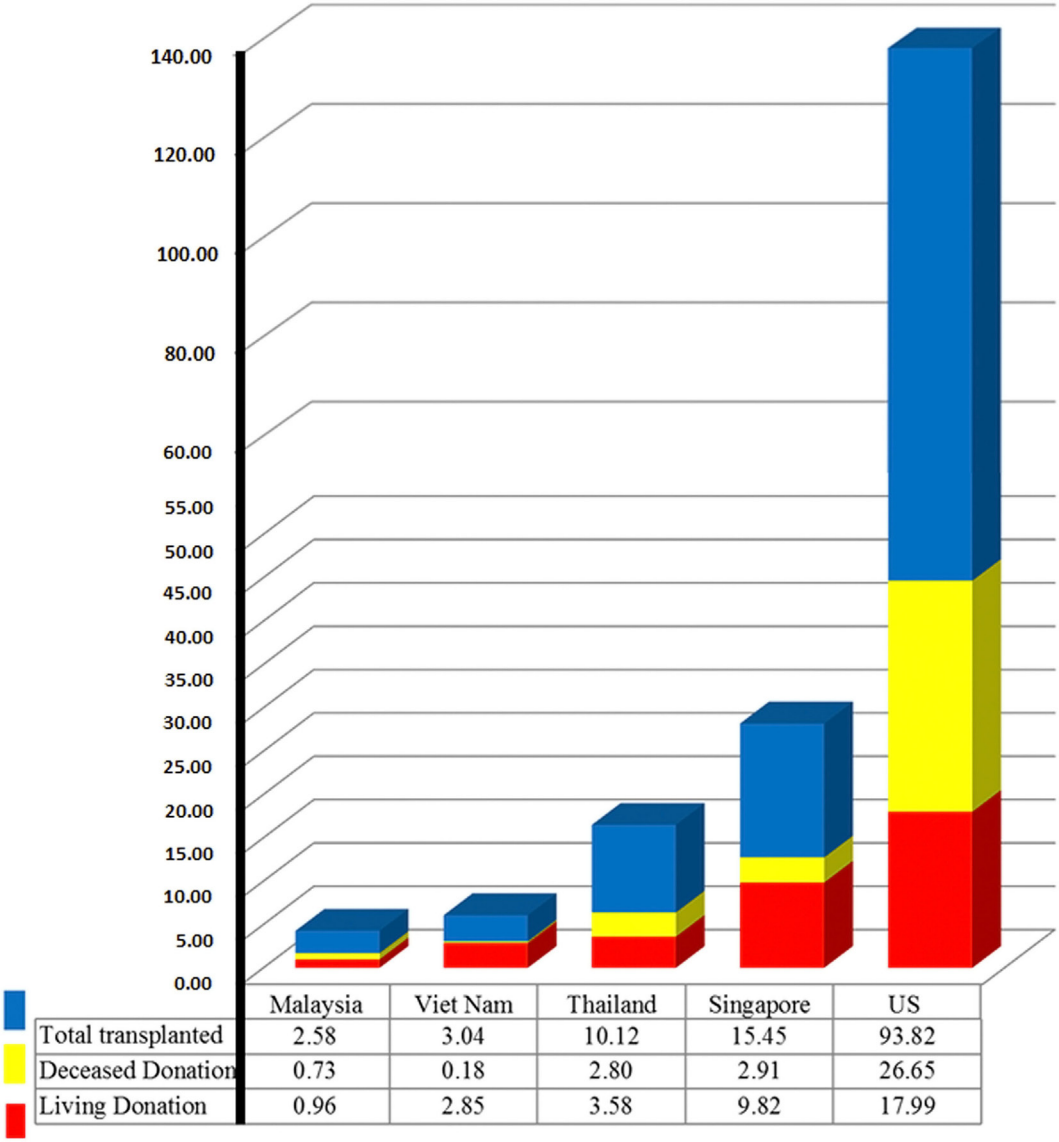
2014 organ donation and transplantation rate in Southeast Asia and the US, patients per million population.

Singapore is fully equipped for serving transplantation needs for its residents. It has a very high HDI and also has the highest incidence of treated ESRD (Figure 2). Though Singapore was not listed above for the largest proportional increase in treated ESRD in the region, as it has already reached a high level of >300 ppmp-treated ESRD in the year 2013, which is close to the figure of 363.1 ppmp-treated ESRD in the US (8). The rate of kidney tx reached a peak at 24.1 ppmp in 2006, but has stayed stable (Figure 2), due to a limitation in the rate of deceased donation. Despite the revision of the organ donation law in 2009, the deceased donation rate runs between 3 and 6 ppmp in Singapore (Table 1; Figure 3), versus a comparison of 25–26 ppmp in the US (Figure 3). Thus, the tx waiting list numbers of patients in Singapore continues to rise.

**Table 1:** 2007–2014 living and deceased donation rates in Southeast Asia, patients per million population.

Countries	2014			2013			2012			2011			2010			2009			2008			2007		
	LD	DD	TT	LD	DD	TT	LD	DD	TT	LD	DD	TT	LD	DD	TT	LD	DD	TT	LD	DD	TT	LD	DD	TT
Brunei Darussalam^a^	NA			NA			NA			NA			NA			5.20	0	5.20	NA			10.5	NA	13.2
Myanmar	NA			0.06	0.02	0.06	0.06	0.02	0.06	0.06	0.02	0.06	0.06	0.02	0.06	0.06	0.02	0.06	0.77	0.02	0.79	0.74	0.02	0.76
Indonesia	NA			1.98	NA	1.98	2.03	NA	2.03	2.05	NA	2.05	2.13	NA	2.13	2.16	NA	2.16	2.12	NA	2.12	2.13	NA	2.13
Malaysia	0.96	0.73	2.58	2.02	0.54	2.83	0.75	0.61	1.98	0.76	0.80	2.53	1.19	0.65	2.54	1.27	0.65	2.76	0.97	0.48	1.93	1.18	0.95	2.40
Vietnam	2.85	0.18	3.04	2.20	0.33	2.53	NA			NA			NA			0.09	NA	0.23	0.09	NA	0.23	0.09	NA	0.23
Philippines	4.01	NA	4.44	2.88	0.37	3.48	3.10	0.45	3.99	3.15	0.45	4.06	2.23	0.51	2.75	4.85	NA	5.55	12.45	NA	12.79	13.00	0.19	13.36
Thailand^b^	3.58	2.80	10.12	4.18	2.36	9.84	3.06	1.95	7.90	3.09	1.63	7.06	3.00	1.28	6.15	2.30	1.28	5.52	3.02	1.26	6.11	3.15	1.42	6.25
Singapore	9.82	2.91	15.45	8.52	4.44	19.07	6.61	2.83	13.77	6.54	3.85	16.92	7.50	5.42	18.75	7.66	5.32	21.06	25.77	6.00	40.44	7.96	5.91	22.27

*^a^Those data based on the WHO-ONT Global Observatory on Donation and Transplantation, except Brunei Darussalam data based on The International Registry in Organ Donation and Transplantation—IRODaT data as of October 4, 2016 (www.irodat.org)*.

*^b^Year 2007 gross number data of Thailand based on Thai Transplant Society and the denominator based on the WHO-ONT Global Observatory on Donation and Transplantation*.

*LD, living donation; DD, deceased donation (actual deceased donor); TT, total organ transplantation; NA; no available data*.

Singapore is the only country in Asia enabling presumed consent under The Human Organ Transplant Act (HOTA), enacted in 1987 (20–22). This act allows for the kidneys of all non-Muslim Singapore citizens and permanent residents between the ages of 21–60 without mental disorders, to be donated in the event of accidental death for tx unless they explicitly opt out. HOTA increased the deceased donor kidney tx rate from an average of 4.7 per annum (approximately 2 ppmp per annum) between 1970 and 1987 to an average of 40.8 per annum (approximately 11.4 ppmp per annum) between 1988 and 2004 (23). To expand the eligible donor pool, HOTA was amended in January 2004 to also allow transplantation of liver, heart, and cornea (24, 25). This bill extended organ donation from donors with non-accidental causes of death and added the regulation for organ donation from living (both related and unrelated) organ donors. It was later amended in August 2008 to include Muslim organ donors, and then in March 2009 to remove the upper age limit for potential deceased donors, paired kidney exchange permission, increased penalties for organ trading, and donor compensation (21, 22). Before the availability of HOTA, the Singapore government utilized the Medical Therapy, Education, and Research Act, which was the opt-in donation law enacted in 1973 (20–22). This law allowed any person to donate organs in the event of death for the purpose of treatment, education, or research (20–22). The Singapore government has also provided the criteria for determining death by brain death or cardiac death under The Interpretation Act (Certification and Determination of Death) (24).

Singapore established the National Organ Transplant Unit in 1970. This body regulates the national organ waiting list and maintains the national registry of recipients and donors (26). To date, kidney, liver, lungs, heart, corneas, skin, bone, and bone marrow tx are available in both public and private hospitals. Living donor tx are performed in both the public and private hospitals while deceased donor tx are carried out only in the public hospitals (27). Singaporeans receiving overseas tx return to follow-up in Singapore and are registered in national renal registry database if they have a functioning tx after 30 days (27).

Singapore has an excellent health financing system, where the public and the private sector work in cooperation for affordable health care (28) that utilizes 4.9% of GDP (Table 2), three times lower than the US, which uses 17.1% of the GDP for health care (15). Singaporean employees contribute to individual medical saving accounts, the Medisave program, which is a national healthcare savings scheme to afford payment for personal and family hospitalization costs. The Ministry of Health subsidizes lower- and middle-income patients for dialysis costs as well as selected immunosuppressive drugs in public healthcare institutions (29). Those patients also use the Medisave benefits to pay for their dialysis or immunosuppressive drugs after the government subsidies (28–30). To protect the poor, low-income patients have a right to apply for and get additional subsidies from the government, and welfare or charity organizations (29), such as the National Kidney Foundation, the Kidney Dialysis Foundation, and the Khoo Foundation.

**Table 2:** Health expenditures, financing policies for dialysis, and transplantation in the transplanted Southeast Asian countries.

Countries	Populations (millions) in 2015	Total spending for healthcare as % of GDP in 2014^a^	Out of pocket payment for health care^b^ (%)	Prevalence of treated end-stage renal disease (ESRD) cases 2013, gross numbers (8)	Prevalence of treated ESRD cases 2013, per million populations (8)	Government policy for dialysis costs coverage	Government coverage for kidney transplantation costs
Singapore	5.6	4.9	54.8	6,955	1,809.1	Minimal	Limited
Brunei Darussalam	0.4	2.6	6.0	NA	NA	100%	100%
Malaysia	30.3	4.2	35.3	33,887	1,140.4	58% of HD versus 100% of PD	100%
Thailand	68.0	6.5	7.9	71,037	1,096.6	– PD first policy group: 100% of PD or 100% of HD if PD contraindicated – Other group: 100% of either PD or HD	100%
Philippines	100.7	4.7	53.7	23,727	224.4	90 sessions HD or 360 days PD per year	NA
Indonesia	257.6	2.8	46.9	NA	65.9	100% HD or 100% of 3-bags per day PD or 80% of 4-bags PD	Partial
Vietnam	93.4	7.1	36.8	NA	NA	100% of either PD or HD	50%
Myanmar	53.9	2.3	50.7	NA	NA	None	None

*^a^Denotes total health expenditure, which is the sum of public and private health expenditure. It covers the provision of health services (preventive and curative), family planning activities, nutrition activities, and emergency aid designated for health but does not include provision of water and sanitation*.

*^b^Denotes out of pocket expenditure, which is any direct outlay by households, including gratuities and in-kind payments, to health practitioners and suppliers of pharmaceuticals, therapeutic appliances, and other goods and services whose primary intent is to contribute to the restoration or enhancement of the health status of individuals or population groups. It is a part of private health expenditure*.

*PD, peritoneal dialysis; HD, hemodialysis; NA, no available data*.

### Tx Outcomes

The unadjusted 1- and 5-year kidney graft survival for living donor are excellent at 98.1 and 95.3%, as reported by the Singapore Renal Registry, compared to 95.1 and 80.2% from the US, as reported by Organ Procurement and Transplantation Network (OPTN) (31, 32). Selection bias and lower patients’ comorbidities are likely causes to support these excellent results. Outcomes for non-heart-beating donor kidney tx outcome are similar to heart-beating donors for the incidence of delayed graft function (50 versus 41%) or 2-year kidney graft survival (96 versus 98%) (33). Paired kidney donation can be undertaken after the last HOTA amendment in 2009 (34). The first case was enrolled in the Paired Exchange Registry in 2014 and operated in early 2015 (34). This program was expected to drive the tx rate, but Singapore’s relatively small population is an obstacle to finding compatible donor–recipient pairs. Hepatitis B virus (HBV)-infected, human immunodeficiency virus-infected patients, and alcoholic patients are excluded from tx in Singapore (26).

## Brunei Darussalam

Brunei Darussalam is an Islamic state that has a high HDI similar to Singapore (13). Bruneians enjoy support from their government, and only 6% of health expenditure comes from personal contributions (15).

### Organ Access, Legislation, and Reimbursement

Dialysis started in Brunei in 1967 and had progressively increased to more than 500 active cases at present (35). Organ tx became available within the country in 2013, with access only to living-related kidney tx. Prior to 2013, eligible ESRD patients were screened and matched with available living-related donors and sent overseas for tx, under government support (35, 36). Despite these efforts, problems exist with transplant tourism. The country has no official cell, tissue, and organ tx law (36), and tx growth in this region is limited due to poor public awareness, cultural concepts about brain death, and poor local transplant facilities (36). Nevertheless, the Brunei government is taking steps to improve access to health and tx care by increasing subsidies for health-care services (37, 38), and the establishment of 15 national health centers (39). It expands access to primary care, with 1.4 physicians per 1,000 people (15) and one nephrologist per 136-treated ESRD patients (38). The first Brunei Dialysis and Transplant Registry was initiated in 2011 (35, 38) and shows the high incidence (265 ppmp) and prevalence (1,289 ppmp) rates of ESRD with the predominant cause of ESRD being due to diabetes (35, 40).

### Tx Outcomes

There have been 49 cases of kidney tx from 1993 to 2012 (36). All of them were performed in foreign centers because the local tx activity only began in 2013. The 5- and 10-year overall graft survival rates are 91.1 and 81.2% (Table 3) with a 10-year patient survival rate of 90%, better than similar reports from Malaysia (41) and the OPTN (42), likely because this cohort of tx Bruneian patients is mostly younger, non-diabetic patients.

**Table 3:** Kidney allograft survival in the major Southeast Asian countries.

Countries	Overall kidney allograft survival			Transplanted year	Reference
	1-year	5-year	10-year		
Singapore	93.2%	86.6%	69.4%	1999–2006	(31)
Brunei Darussalam^a^	NA	91.1%	81.2%	1993–2012	(36)
Malaysia	93%	83%	67%	2005–2014	(46)
Thailand^b^	96.2%	90.0%	78.9%	2002–2006	(63)
Vietnam^c^	82%	NA	NA	2004–2008	(104)
Myanmar^c^	95%	NA	NA	1997–2003	(107)

*^a^Living donor graft from overseas from the national registry*.

*^b^Deceased donor graft from the national registry*.

*^c^Living donor graft reported from the single center*.

*NA, no available data*.

## Malaysia

Malaysia is a multicultural and multi-faith country, consisting of Muslims, Buddhists, Christians, and Hindus, with Malay, Chinese, and Indian ethnicity (43). It has pursued economic growth and reached upper middle income (44). Malaysia has a high HDI, above the average Southeastern Asia HDI (13) and it spends 4.2% of its GDP in health expenditure (15).

### Organ Access, Legislation, and Reimbursement

Renal replacement therapy was introduced in Malaysia in 1976 (45). Malaysia has an active dialysis program, with about 758 dialysis centers, most (94%) offering hemodialysis (46). Due to increased acceptance of dialysis as a mainstay therapy for ESRD in Malaysia, the prevalence of dialysis increased from 13,000 in 2005 to 35,000 in 2014 (46) (Figure 2). Malaysia is a welfare-oriented state (47). The Malaysian government is the principal payer of renal replacement therapy by a public–private partnership model (48, 49), and the government subsidizes both public dialysis centers and non-governmental organizations (48) such as the National Kidney Foundation. The Private Healthcare Facilities and Services Act, enacted in 2006, allowed hemodialysis facilities to be set up outside hospitals areas, which resulted in the rapid growth of private dialysis units and increased hemodialysis access (46, 48, 49). Tx was initially performed in Malaysia in the 1970s (45). Malaysia provides kidney, liver, and lung tx mostly in public medical centers under the National Organ, Tissue, and Cell Transplantation Policy, which was enacted in 2007 (50, 51). Organ tx programs are overseen by the National Transplant Council (51). The National Transplant Procurement Management Unit helps with deceased donor allocation and is supported by the local Tissue Organ Procurement Team in each donor hospital (51). Brain death diagnosis and certification is done under guidance from the Brain Death Committee (52). The National Transplant Waiting list is managed by The National Transplantation Unit, which was established within the Ministry of Health for regulating financial support and monitoring ethical standards for tx (51, 53). Between 2005 and 2014, the kidney tx rates fell by 50%, from 6 to 3 ppmp (46) due to a reduction in tx tourism, mainly in China (47) due in significant part to increased local legislation and the Istanbul Declaration (54), which desist organ trafficking. Living donation has to be authorized by the Unrelated Transplant Approval Committee (51) to limit transplant tourism.

### Tx Outcomes

According to the 2014 Malaysian Dialysis and Transplant Registry, the overall renal graft survival rates are 93, 88, 83, and 67% in year 1, 3, 5, and 10, respectively (Table 3) (46). The primary immunosuppressive regimen used are based on calcineurin inhibitor-based therapy with a decreasing use of cyclosporine (59% in 2010 to 44% in 2014) and increasing use of tacrolimus (32% in 2010 to 45% in 2014). Mycophenolic acid is used for 68% of kidney tx as maintenance therapy (55).

## Thailand

Thailand has a lower HDI than the previous regions and spends 6.5% of its GDP on health care (15). 7.9% of Thai health expenditure comes from personal contributions (15). Physician coverage in Thailand is sparse with 0.4 physicians per thousand people (15), much lower than Malaysia (1.2 physicians per thousand people) or Singapore (2 physicians per thousand people), even though the incidence of treated ESRD is equivalent (Figure 2).

### Organ Access, Legislation, and Reimbursement

The growing incidence and prevalence of ESRD (157.8% increase from 2007 to 2009), with stable numbers of kidney tx in the region (9), has contributed to an expanding kidney tx waiting list in Thailand. The Universal Coverage Scheme (UCS) authorized by the National Health Security Act enacted in 2002 (56–58), supplemented health-care support (56–59) with expanded coverage for renal replacement therapy through the Thai peritoneal dialysis (PD) first policy (2007) and kidney tx (9) (Table 2). To date, all Thais can get access to dialysis and tx through one of three major health schemes in the country, the largest being through UCS (9, 60).

The first kidney tx was done in Thailand in 1972 (61, 62). Thailand now provides bone marrow, liver, heart, lung, and heart and lung transplantations in 26 transplant centers around the country (63). Brain death criteria were set up in 1989 and revised in 2011 (64). The Organ Donation Center is a non-for-profit, central organization, established in 1994 under the umbrella of the Thai Red Cross Society (64, 65), responsible for national waiting lists, centralized deceased donor allocation, accreditation of tx centers, with voluntary data reporting from tx centers (64). Public organ donation campaigns have been supported by the Thai Royal family to increase awareness, organ access, and extended coverage for kidney tx (65, 66). Moreover, the Organ Donation Center with collaboration from the Ministry of Public Health have established full-time transplant coordinator teams in 24 referral hospitals to help with organ recovery and procurement (67). As a result, the kidney tx rate is increasing (Figures 1 and 2).

### Tx Outcomes

The renal graft survival and patient survival rates are comparable to other registries and have improved significantly over the last two decades (63). One-, 5-, and 10-year death-censored renal graft survival rates are 95.4, 89.3, and 83.7% for living donor tx versus 96.2, 90.0, and 78.9% for deceased donor tx (63). The most commonly used immunosuppressive regimen comprises of tacrolimus, mycophenolate, and prednisolone that is similar to the US. Induction therapy is used for 70.4%; interleukin-2 receptor antagonist is the most common induction agent while T-cell-depleting agents are more common in the US (68). Induction therapy is being increasingly used, with 55% use in 2012 to 70.4% in 2015 (63, 68, 69). Pediatric kidney tx outcomes reported 1-year patient and graft survival rates of 100% either for living donor or deceased donor tx, with similar immunosuppressive regimens as in adults. One-, 5-, and 10-year graft survival rates are 96.2, 81.9, and 64.4%, respectively (68). Thailand offers kidney tx for HBV- and HCV-positive patients, as well as ABO-incompatible kidney tx in some centers (70). Donation after cardiac death and paired kidney exchange program is not available to date.

## Philippines

The Philippines spends 4.7% of GDP for health expenditure, but unlike the former countries that have more government support (Table 2), Filipinos personally pay for about 53.7% of their health expenses (15).

### Organ Access, Legislation, and Reimbursement

The Philippines have growing rates of ESRD (Figure 2), with expanding wait lists as organ donation rates are stagnant with deceased donation rate as low as 1 ppmp (Table 1) (71). The flat rate is on the grounds of high out-of-pocket in health-care costs for dialysis and living kidney tx (72), and a shortage of the necessary infrastructure support to enhance organ donation, procurement, allocation, and tx (73). The Philippine Health Insurance Corporation (PhilHealth) was implemented in 1995, but partial coverage for dialysis was only included in an amendment in 2006 (74, 75). In 2012, PhilHealth provided the Z Benefits Package covering catastrophic illnesses like malignancies and kidney tx, and in 2015, it expanded coverage for hemodialysis sessions from 45 to 90 sessions per year, and for PD from 270 to 360 days per year (76). The Philippines performs kidney, liver, pancreas, bone marrow, and islet cell as well as double transplants like liver and kidney or pancreas and kidney (77, 78). The Philippine Network for Organ Sharing (PHILNOS), established by the Department of Health in 2010, is the central coordinating body of all deceased organ or tissue donation and tx activities (79, 80) and governs various operational guidelines including the brain death protocol, allocation scoring, deceased donor referral, waiting lists. The PHILNOS is monitored by the External Audit Department with particular emphasis on organ allocation procedures. The PHILNOS runs the Philippines Organ Donor and Recipient Registry System, and the deceased allocation system is centralized under the Donor Allocation Scoring System (81). The National Transplant Advisory Board, National Transplant Ethics Committee, and the Kidney Donor Monitoring Unit, all help set tx guidelines (82).

Kidney tx in the Philippines is mostly from living donors (94%) (Table 1), from both living-related and unrelated donors; with the larger proportion (70.1%) being from unrelated donors (83). For related donors, non-immediate family members are also eligible donors (73). Donor compensation as “gratitudinal gifts” to living unrelated donor is allowed (82). Before 2008, the government approved a 10% cap to each tx center to allocate living donors from Filipinos to foreigners, in situations when Filipinos recipients were not available (82, 84–86). Unfortunately, this resulted in unacceptable rates of organ trafficking such that, in 2008, in an effort to eliminate tx tourism, the PHILNOS prohibited all tx to foreigners using Filipino organs. The World Health Organization identified the Philippines, China, and Pakistan as key areas driving organ trafficking (80). With support from the 2008 Declaration of Istanbul on Organ Trafficking and Transplant Tourism, the Philippines government enacted the anti-commercialization of human organs, tissues, or part of living persons Act of 2008 (86, 87). This Act established the Philippines Board for Organ Donation and Transplantation to oversee policy implementation and to define penalties by imprisonment and fines.

### Tx Outcomes

The majority of kidney tx patients in the Philippines are prescribed tacrolimus, mycophenolate, and prednisone for initial immunosuppression. As most of them are living donor recipients, 99% of recipients received induction therapy with either Basiliximab or rabbit anti-thymocyte globulin, and 98% have immediate graft function, with minimal or absent rates of delayed graft function in most centers (88). Nevertheless, the report of graft and patient survival rates from the Philippines are difficult to access.

## Indonesia

Indonesia is the most populous Muslim-majority in the world with the largest economy in Southeast Asia (89). Though the government had expanded universal coverage for nearly half of population in 2014, Indonesians still pay out-of-pocket for 46.9% of health expenditure (Table 2) (15, 90). The little government spending in social health security increases the health-care cost burden on patients and families (91). This impact is most on the poor, who can have limited access to dialysis and tx care.

### Organ Access, Legislation, and Reimbursement

Hemodialysis was introduced in Indonesia in 1967 and PD in 1985, but both have progressed slowly due to limited reimbursement (92, 93). After the government initiated health-care reform with dialysis coverage in 2005, the number of treated ESRD has grown rapidly to ~200 ppmp by 2013 (8). However, detailed data on the actual gap between patients needing dialysis and those receiving kidney transplants in the region are lacking (48), and it seems that up to one-third of ESRD treatment may be self-funded (30).

The first kidney tx in Indonesia was recorded in 1977 (94). The Indonesia 1991 Health Regulation provided for the procurement of tissue from living donors, but not from deceased donors (95). The Kemayoran Consensus was signed in 1995, which stated that all religions in Indonesia accepted the concept of organ tx, and human organ commercialization is forbidden (94). In support of this, the Indonesia Islamic Groups and the Federation of the Islamic Medical Association have endorsed the concepts of brain death (52). In 2014 (89–91, 96), the government launched Indonesia’ universal health coverage/jaminan kesehatan nasional (UHC/JKN) to cover 120 million people who are already engaged in various social health insurance schemes into the Social Security Management Agency for the Health Sector (89). The UHC/JKN program has a target to enroll 250 million people by 2019.

For those included in this program, all costs for hemodialysis and PD with three continuous ambulatory PD fluid exchanges are covered by government health insurance (93). Costs for kidney tx are also covered partly by government health insurance (97), though to date, only 7 from 33 medical centers in the country participate for full JKN coverage (90). There are concerns that the growth of JKN may be halted by regulation disparities, poor health-care infrastructure, and the urban–rural gap in the number of medical staff (90).

### Tx Outcomes

In 2009, three centers in Indonesia performed kidney tx. Kidney tx from “emotionally-related” donors and the spouse, is accepted in Indonesia (94). Five- and 15-year renal graft survival rates are 60 and 50% (94). Immunosuppressive drug combinations consist of either cyclosporine A, azathioprine, and corticosteroids or tacrolimus, mycophenolate mofetil, and corticosteroids (97).

## Vietnam

Vietnam has enjoyed rapid economic growth with a dramatic reduction in poverty over the last decade (98). Social and health-care outcomes have improved significantly (98). In 2014, the Vietnamese government spent 7.1% of its GDP on health care and Vietnamese personally pay for ~36.8% of health-care expenditure (Table 2) (15).

### Organ Access, Legislation, and Reimbursement

Major economic reforms have been introduced in Vietnam including the introduction of social health insurance and permission for private sectors in health care. Dialysis was included in insurance coverage with erythropoietin therapy in 2005, and ~50% of tx therapy is covered (99–101). Increasing numbers of patients receive hemodialysis and PD in Vietnam, and it is estimated that the prevalence of treated ESRD is more than 120 ppmp (100). The first kidney tx was performed successfully in Vietnam in 1992 (99), and currently, Vietnam also performs liver and heart transplants. Before 2006, only living-related organ tx were allowed. The Vietnamese government passed a law in 2006 for regulation of living and deceased organ donation, the organization of tissue banks, the establishment of the National Coordination Center for Human Organ Transplantation, and decreeing prohibiting of organ commercialization (99, 102). The law provided living donor compensation such as free health insurance cards, free regular health check, and to get prioritized tx themselves. It also addressed brain death criteria, diagnostic protocols, and the establishment of tx centers. Living donors had to have the same bloodline with the recipient or were required to be the next of kin within three generations of the recipient. The law also allowed Vietnamese in going abroad for donating organs to their relatives or vice versa (102). Unrelated living donors, namely, husbands or wives or friends were acceptable donors.

The National Coordination Center for Human Organ Transplantation is responsible for organizing donated body tissue and organs and will cooperate with tissue banks and hospitals nationwide. To date, ~400 living donor kidney and liver txs and ~10 deceased donor txs have been done in Vietnam. Thirteen health-care facilities meet the regulations and conditions for carrying out body tissue and organ txs in Vietnam (103). The deceased donor organ tx program still faces impediments and suggested improvements to the system are to set up a national online database system and increased public education on deceased donor donation.

### Tx Outcomes

Single center data show that 1- and 3-year renal graft survival is 82 and 65% from living donors, with 1- and 3-year patient survival at 82 and 73%, respectively (Table 3) (104). Immunosuppression usage consists of anti-interleukin-2 receptor monoclonal antibody induction and triple drug regimen as maintenance therapy with cyclosporine or tacrolimus, mycophenolate mofetil or mycophenolic acid sodium, and corticosteroids.

## Myanmar

### Organ Access, Legislation, and Reimbursement

There is the lack of government health-care payment for chronic disease care like dialysis or tx in Myanmar (105). Burmese patients support high out-of-pocket costs to the tune of 50% of their health-care expenditures (Table 2) (15). Recently, a trial prepaid health insurance system was started in July 2015 with cooperation from national and international non-governmental organizations (106).

The first kidney tx in Burma was done in 1997 (107). Only two hospitals in Myanmar perform kidney txs—Yangon General Hospital and Mingalardon Military Hospital (108). Due to a lack of sufficient infrastructure to support tx needs for all patients with ESRD in Myanmar, many private-pay-patients get their tx in India (109). The government promulgated the Body Organ Donation Law in 2004, which allows deceased organ donation and living-related organ donation within immediate family members (108, 110). This law sets heavy penalties for organ commercialization by imprisonment for up to 3 years.

### Tx Outcomes

There are limited sources of information to find out the number and clinical outcome of tx activities in Myanmar. However, there is a single-center report of 21 living-related donor kidney tx with 95% 1-year- and 91% 6-year-graft survival rates (Table 3) (107).

## Laos, Cambodia, and Timor-Leste

Cambodia has at least 10 hemodialysis units available for ~200 patients, but patients have to cover 100% of costs as there is no government subsidy (111). To date, there are no significant tx activities in Laos, Cambodia, and Timor-Leste, and patients have to go overseas for tx.

There is a growing demand for organ tx in Southeast Asia, which is currently not being met due to various reasons that range from access, infrastructure, economics, and legislation. The unmet need is evident, but ESRD disease epidemiology and prevalence can be difficult to assess in some countries in Southeast Asia due to a lack of standardized national registry data. Improved access to tx will also require increased procedure affordability by transitioning health-care financing away from the patient and toward national coverage (9). The WHO Madrid consultation (10, 112) in 2010 proposed a 1–5 tier system (with 5 being the highest) for ranking the capabilities of regional tx services. Three of the 11 Southeast Asian countries are at level 1 (Laos, Cambodia, and Timor-Leste). Thailand and Singapore were reported to have level 4 services, Malaysia, the Philippines, and Vietnam have level three services, and Indonesia and Myanmar showed having level 2.

## Suggested Strategies for Increasing Tx in Southeast Asia

A review of the regions above suggests that improved access and outcomes for ESRD and tx will require attention to the following prime factors.

### Developing a Framework to Increase Deceased Donation

There are two deceased donor structures in the Southeast Asia, i.e., the opting-in system based on informed consent, and the opting-out system based on presumed consent. Singapore is the only state in the region that has the opting-out scheme. Enrollment for the opting-in scheme is hampered in regions that have low rates of available physicians and trained coordinators, and high clinical services workload can lead to less time for communication. *A priori* consent at information repository centers such as driver licensing registration or tax-return registration can improve donor organ access.

Southeast Asia has a high incidence of deaths from fatalities involving stroke and road traffic accidents. In fact, Thailand ranks second in the world for traffic fatalities with a mortality rate of 36.2 patients per 100,000 population (113) and fatality from stroke is the most common cause of death in Thailand with more than 250,000 new cases and 50,000 lives claimed annually (114). Thus, there can be a high volume of eligible brain death donors. Legislation to support the diagnosis of brain death and optimal support from transplant coordinator teams is crucial to increase the deceased donation rate in this region. Another measure to expand the donor pool is to enable the use of non-heart-beating donors as outcomes for kidney grafts are comparable to transplants from brain dead donors in the US and Singapore (33, 115). Education of professionals with organ reperfusion skills to maintain these organs is also needed in the ICUs in these regions (115–117).

### Increased Access to Living Donors

Increased rates of living donation can be accomplished by inclusion of unrelated donors, namely emotionally related donors, such as a spouse, friend, in-law, stepparent, adoptive children, friend, or even an altruistic stranger (118, 119). But concerns lie in this region that expanding the criteria for acceptable living donors can adversely impact organ commercialism (73, 84, 120). Southeast Asian countries allow living donation from a spouse included Malaysia, Thailand, Philippines, Indonesia, and Vietnam. In Myanmar, only immediate family members are authorized to donate.

### Donor Compensation—The Sticky Wicket

Donor “compensation” is interpreted as donor “incentives” by some. The term “incentive” refers to all forms of material gain or similar advantage offered for consent to living donation or authorization of deceased donation of organs (121). The term “compensation” refers to payment for the costs incurred around tx that would cause the donor financial hardships and would include activities such as traveling and accommodation costs and lost wages (122–124). In the US, National Organ Transplant Act has allowed for financial compensation for the necessary expenses incurred by the donor of a human organ (125). In 2004, the Organ Donation and Recovery Improvement Act was launched to enable funding for an organ donation awareness program and public education as well as funding for donor compensation (126). The National Living Donor Assistance Center approves of grants to pay for travel and subsistence expense for some living donors who have financial hardship (125). In countries without universal health care, prospective donors might not have insurance to cover following complications or even to pay for a routine check-up. Often, potential donors are also concerned that donation might restrict their hiring opportunities (127). The Philippines program has allowed gifts based on gratitude, supporting compensation for health and life insurance, lost income, and education (82, 84). Regulated compensation can provide a rational, accessible, and equitable donor allocation program (82). Aside from financial compensation, non-financial support for the donor can come in the form of community recognition that is a valuable commodity in Asia. Saudi Arabia offers donors “the King Abdul Aziz medal” as well as a discount card with Saudi Airlines (128).

### Management of the National Registry, Waiting Lists, and Central Allocation

Professional organizations such as the nephrology societies in each region should support national registries; the Singapore Renal Registry, the Malaysian National Renal Registry, and Thailand Renal Replacement Therapy Registry are three such registries in the area. A centralization of deceased organ allocation will ensure equity of allocation and get the best match.

### Health Financing System

We think that health-care reform can help increase end-stage organ disease access to treatment and tx activities in this region. Aside from Medicare and Medicaid that enacted since 1965, Americans have abided by the Patient Protection and Affordable Care Act that passed in 2010 (129). The last law mandates that persons over the age of 18 obtain health insurance, either through their employer or public insurance program in case they cannot afford insurance otherwise. Having a reliable funding for health care is mandated to improve access to renal replacement therapies.

### Education

The tx workforce in Asia is very limited (130). Without strategic planning to improve the number of well-trained specialists, the delivery of care to patients with end-organ failure or tx will be threatened (130). Education of the population is also critical, especially in a region with many religious, cultural, and social traditions, which can create barriers to organ donation (131, 132). The drive for altruistic donation in the community requires continued public awareness and public education.

In conclusion, there is recognition of the growing burden of end-stage organ failure in Southeast Asia. The gap between the tx waiting lists and available organs for tx is expanding.

Improved legislation, health-care reform, and education are key drivers to improve donor availability. National registries are urgently needed in the entire region to review and improve tx access and outcomes. Continued heightened surveillance to support legal practices in organ harvesting and donation and strict laws to curb transplant commercialization and tourism are necessary to have in place and enforce. With these practices in place, the next decade will likely show rapid advances in access to state of the art medical management for renal failure and renal tx, with a significant impact on improving population mortality and morbidity in Southeast Asia.

## Author Contributions

This review is conducted under the conception and design from MS and CC. Reviewing relevant evidence and data analysis is done by CC with final correction and approval by MS.

## Conflict of Interest Statement

The authors declare that the research was conducted in the absence of any commercial or financial relationships that could be construed as a potential conflict of interest.

## References

[1] Merrill JP Murray JE Harrison JH Successful homotransplantation of the human kidney between identical twins. J Am Med Assoc (1956) 160(4):277–82. 10.1001/jama.1956.02960390027008 13278189

[2] Starzl TE The landmark identical twin case. J Am Med Assoc (1984) 251(19):2572–3. 10.1001/jama.251.19.2572 PMC2993573 6371267

[3] Medin C Elinder CG Hylander B Blom B Wilczek H. Survival of patients who have been on a waiting list for renal transplantation. Nephrol Dial Transplant (2000) 15(5):701–4. 10.1093/ndt/15.5.701 10809814

[4] Wetmore JB Collins AJ Global challenges posed by the growth of end-stage renal disease. Ren Replace Ther (2016) 2(1):15 10.1186/s41100-016-0021-7

[5] Tamura MK. Incidence, management, and outcomes of end-stage renal disease in the elderly. Curr Opin Nephrol Hypertens (2009) 18(3):252–7. 10.1097/MNH.0b013e328326f3ac 19374012 PMC2738843

[6] Organ Donation and Transplantation Activities 2014. The Global Observatory on Donation and Transplantation (GODT) Data. The WHO-ONT Collaboration (2016). Available from: http://www.transplant-observatory.org/data-reports-2014/

[7] Shimazono Y. The state of the international organ trade: a provisional picture based on integration of available information. Bull World Health Organ (2007) 85(12):955–62. 10.2471/BLT.06.039370 18278256 PMC2636295

[8] United States Renal Data System. 2015 USRDS Annual Data Report: Epidemiology of Kidney Disease in the United States. Bethesda, MD: National Institutes of Health, National Institute of Diabetes and Digestive and Kidney Diseases (2015).

[9] Praditpornsilpa K Lekhyananda S Premasathian N Kingwatanakul P Lumpaopong A Gojaseni P Prevalence trend of renal replacement therapy in Thailand: impact of health economics policy. J Med Assoc Thai (2011) 94(Suppl 4):S1–6. 22043559

[10] White SL Hirth R Mahíllo B Domínguez-Gil B Delmonico FL Noel L. The global diffusion of organ transplantation: trends, drivers and policy implications. Bull World Health Organ (2014) 92(11):826–35. 10.2471/BLT.14.137653 25378744 PMC4221768

[11] World Bank. World Bank Country and Lending Groups. Washington, DC (2016). Available from: https://datahelpdesk.worldbank.org/knowledgebase/articles/906519-world-bank-country-and-lending-groups

[12] United Nations, UN Development Programme (UNDP). Human Development Report 2013 – The Rise of the South: Human Progress in a Diverse World. (2013). Available from: http://www.refworld.org/docid/514850672.html

[13] United Nations, UN Development Programme (UNDP). Human Development Report 2015 – Work for Human Development. (2016). Available from: http://report.hdr.undp.org

[14] Nielsen L Classifications of Countries Based on Their Level of Development: How It Is Done and How It Could Be Done. IMF Working Papers (2011). p. 1–45. Available from: https://ssrn.com/abstract=1755448

[15] World Bank. World Development Indicators. Washington, DC (2016). Available from: http://wdi.worldbank.org/table/2.15#

[16] United Nations, United Nations Statistic Division. Composition of Macro Geographical (Continental) Regions, Geographical Sub-Regions, and Selected Economic and Other Groupings. New York, NY (2016). Available from: http://unstats.un.org/unsd/methods/m49/m49regin.htm#asia

[17] Hooi LS Wong HS Morad Z. Prevention of renal failure: the Malaysian experience. Kidney Int Suppl (2005) 94:S70–4. 10.1111/j.1523-1755.2005.09418.x 15752245 PMC7112354

[18] Lim YN Lim TO Lee DG Wong HS Ong LM Shaariah W A report of the Malaysian dialysis registry of the National Renal Registry, Malaysia. Med J Malaysia (2008) 63(Suppl C):5–8. 19230240

[19] Population Reference Bureau. Average Life Expectancy in Asia for Those Born in 2015, by Gender and Region (in Years). New York, NY (2016). Available from: https://www.statista.com/statistics/274516/life-expectancy-in-asia/

[20] Chin JJ Kwok TH After presumed consent: a review of organ donation in Singapore. Indian J Med Ethics (2014) 11(3):139–43. 25160963 10.20529/IJME.2014.038

[21] Singapore Government, Ministry of Health. The Human Organ Transplant Act, Legislative Acts and Guidelines. Singapore (2016). Available from: https://www.moh.gov.sg/content/moh_web/home/legislation/legislation_and_guidelines/human_organ_transplantact.html

[22] Singapore Government, Ministry of Health. The Human Organ Transplant Act. Singapore (2016). Available from: https://www.liveon.sg/content/moh_liveon/en/organdonation/hota.html

[23] Shum E Chern A. Amendment of the human organ transplant act. Ann Acad Med Singapore (2006) 35(6):428–32. 16865196

[24] Singapore Government, Attorney-General’s Chambers. The Interpretation (Determination and Certification of Death) Regulations. Singapore (2016). Available from: http://statutes.agc.gov.sg/aol/search/display/view.w3p;query=CompId%3Ae3c7f456-5adc-4fec-a6d8-59fdeba22902%20ValidTime%3A20150101000000%20TransactionTime%3A99991231000000;rec=0#pr1-he-

[25] Kwek TK Lew TW Tan HL Kong S. The transplantable organ shortage in Singapore: has implementation of presumed consent to organ donation made a difference? Ann Acad Med Singapore (2009) 38(4):346–8. 19434338

[26] Schmidt VH Lim CH. Organ transplantation in Singapore: history, problems, and policies. Soc Sci Med (2004) 59(10):2173–82. 10.1016/j.socscimed.2004.03.014 15351482

[27] Ministry of Health, National Registry of Diseases Office HPB. Singapore Renal Registry Report No.9 Trends in Chronic Kidney Failure in Singapore 2010-2011. Singapore (2016). Available from: https://www.nrdo.gov.sg/docs/librariesprovider3/Publications---Kidney-Failure/trendsinrenal2010-2011.pdf?sfvrsn=0

[28] World Bank Group. Financing Health Care Singapore’s Innovative Approach. Washington, DC (2016). Available from: https://openknowledge.worldbank.org/handle/10986/11299

[29] Singapore government, Ministry of Health. Costs and Financing. Singapore (2016). Available from: https://www.moh.gov.sg/content/moh_web/home/costs_and_financing.html

[30] Morad Z Choong HL Tungsanga K Suhardjono Funding renal replacement therapy in Southeast Asia: building public-private partnerships in Singapore, Malaysia, Thailand, and Indonesia. Am J Kidney Dis (2015) 65(5):799–805. 10.1053/j.ajkd.2014.09.031 25736214

[31] Vathsala A Chow KY Renal transplantation in Singapore. Ann Acad Med Singapore (2009) 38(4):291–9. 19434331

[32] Ministry of Health, National Registry of Diseases Office HPB. Singapore Renal Registry Annual Registry Report 1999–2013 (Preliminary). Singapore (2016). Available from: https://www.google.com/search?q=Singapore+Renal+Registry+Annual+Registry+Report+1999%E2%80%932013+(Preliminary).+2014.&oq=Singapore+Renal+Registry+Annual+Registry+Report+1999%E2%80%932013+(Preliminary).+2014.&aqs=chrome.0.69i57.907j0j8&sourceid=chrome&ie=UTF-8

[33] Lau KO Vathsala A Kong S Li MK Preliminary results of heart-beating and non-heart-beating donor kidney transplants – the Singapore experience. Ann Acad Med Singapore (1999) 28(2):222–6. 10497671

[34] Matthews H First Living Paired Kidney Exchange in Singapore Performed at NUH. News Asia (2016). Available from: http://www.channelnewsasia.com/news/singapore/first-living-paired/2558730.html

[35] Tan J. Renal replacement therapy in Brunei Darussalam: comparing standards with international renal registries. Nephrology (2014) 19(5):288–95. 10.1111/nep.12228 24641721

[36] Tan J Khalil MAM Tan SY Khalil M Ahmed D Zinna S Outcomes of renal transplantation in Brunei Darussalam over a twenty-year period (1993–2012). J Transplant (2014) 2014:784805 10.1155/2014/784805 25478205 PMC4247949

[37] Commonwealth Secretariat, Commonwealth Secretariat’s Social Transformation Programmes Division. Health Systems in Brunei Darussalam. London (2016). Available from: http://www.commonwealthhealth.org/asia/brunei_darussalam/health_systems_in_brunei_darussalam/

[38] Tan J End stage renal disease in Brunei Darussalam – report from the first Brunei Dialysis Transplant Registry (BDTR). Ren Fail (2013) 35(8):1101–4. 10.3109/0886022X.2013.815101 23879396

[39] Tant EM Equity in Access to Healthcare in Brunei Darussalam: Results from the Brunei Darussalam Health System Survey (HSS). [Master’s Thesis]. Durham: Global Health Institute, Duke University (2014). 66 p.

[40] The International Food Policy Research Institute. 2015 Nutrition Country Profiles in global Nutrition Report. Washington, DC (2016). Available from: http://globalnutritionreport.org/the-data/nutrition-country-profiles/

[41] National Renal Registry (Malaysian Society of Nephrology). Malaysian Dialysis and Transplant Registry. Kuala Lumpur (2016). Available from: http://www.msn.org.my/

[42] Freitas MC Kidney transplantation in the US: an analysis of the OPTN/UNOS registry. Clin Transpl (2011):1–16. 22755397

[43] Department of Statistics Malaysia, Official Portal. Population Distribution and Basic Demographic Characteristic Report 2010. Putrajaya (2017). Available from: https://www.dosm.gov.my/v1/index.php?r=column/ctheme&menu_id=L0pheU43NWJwRWVSZklWdzQ4TlhUUT09&bul_id=MDMxdHZjWTk1SjFzTzNkRXYzcVZjdz09

[44] World Bank Group. Malaysia Overview. Washington, DC (2016). Available from: http://www.worldbank.org/en/country/malaysia/overview

[45] Salwani F A Socio-Legal Study on Organ Shortage in Malaysia. [Doctoral Thesis]. Southampton: ePrints Soton, University of Southampton, School of Law (2012). 346 p.

[46] Goh BL Ong LM, editors. Twenty Second Report of the Malaysian Dialysis and Transplant 2014. Kuala Lumpur: The National Renal Registry, Malaysian Society of Nephrology (2015).

[47] Barraclough S. The growth of corporate private hospitals in Malaysia: policy contradictions in health system pluralism. Int J Health Serv (1997) 27(4):643–59. 10.2190/NTFT-QRBY-6VAJ-FMM9 9399111

[48] Prasad N Jha V Hemodialysis in Asia. Kidney Dis (Basel) (2015) 1(3):165–77. 10.1159/000441816 27536677 PMC4934815

[49] Nik Rosnah WA Lee KH Impact of the private healthcare facilities and services act 1998 (act 586) & regulations 2006 on the medical practice in corporate private hospitals in Malaysia. OIDA Int J Sustain Dev (2011) 2(9):89–106.

[50] World Health Organization, World Health Organization Regional Office for the Western Pacific. World Health Organization Guiding Principles on Human Organ Transplantation. Report of the Regional Meeting. Kuala Lumpur (2009). Available from: http://www.wpro.who.int/health_technology/documents/docs/HumanOrganTransplantationMeetingReport.pdf?ua=1

[51] Ministry of Health Malaysia, Medical development Division. The National Organ Tissue and Cell Transplantation Policy. Putrajaya (2016). Available from: http://www.moh.gov.my/images/gallery/Polisi/National_Organ_Tissue_Cell_Transplan_Policy.pdf

[52] Ministry of Health, Academy of Medicine of Malaysia and the Malaysian Society of Neurosciences. The Consensus Statement on Brain Death 2003, for Details on Procedures and Technical Instructions. Malaysia (2016). Available from: http://www.moh.gov.my/images/gallery/orga/Consensus%20statement%20on%20brain%20death%202003.pdf

[53] Morales Pedraza J Ethical Policy and Principles in Tissue Banking. Switzerland: Springer International Publishing (2016). 133 p.

[54] The Declaration of Istanbul on Organ Trafficking and Transplant Tourism. Istanbul summit. Nephrol Dial Transplant (2008) 23(11):3375–80. 10.1093/ndt/gfn553 18922861

[55] Goh BL, editor. Eleventh Report of the National Transplant Registry 2014. Kuala Lumpur: The National Renal Registry (2016).

[56] Antos JR Taylor WH Health Care Financing in Thailand: Modeling and Sustainability. Report from the Workshop on Model Development of Sustainable Health Care Financing. Bangkok (2016). Available from: http://siteresources.worldbank.org/INTTHAILAND/Resources/333200-1182421904101/2007aug-health-financing-modeling.pdf

[57] Hanvoravongchai P Thailand – Health Financing Reform in Thailand: Toward Universal Coverage Under Fiscal Constraints. UNICO Study Series; No. 20. Washington, DC: World Bank (2013). Available from: http://documents.worldbank.org/curated/en/476621468132279566/Thailand-Health-financing-reform-in-Thailand-toward-universal-coverage-under-fiscal-constraints

[58] Towse A Mills A Tangcharoensathien V Learning from Thailand’s health reforms. BMJ (2004) 328(7431):103–5. 10.1136/bmj.328.7431.103 14715608 PMC314057

[59] Tangcharoensathien V Pitayarangsarit S Patcharanarumol W Prakongsai P Sumalee H Tosanguan J Promoting universal financial protection: how the Thai universal coverage scheme was designed to ensure equity. Health Res Policy Syst (2013) 11:25. 10.1186/1478-4505-11-25 23919275 PMC3735425

[60] Anutrakulchai S Mairiang P Pongskul C Thepsuthammarat K Chan-on C Thinkhamrop B. Mortality and treatment costs of hospitalized chronic kidney disease patients between the three major health insurance schemes in Thailand. BMC Health Serv Res (2016) 16:528. 10.1186/s12913-016-1792-9 27686066 PMC5043539

[61] Benyajati C Pochanugool C Sitprija V Suvannapha R Bunyaratavej P Satitpunwaycha P Early experience in renal transplantation in Thailand. J Med Assoc Thai (1973) 56:398–403. 4618562

[62] Sumethkul V Jirasiritham S Chiewsilp P Domrongkitchaiporn S Sujirachato K Mongkolsuk T The organization of Kidney Transplantation Services at Ramathibodi hospital: fourteen years experience on waiting list, kidney donors and kidney transplantation. J Med Assoc Thai (2000) 83(Suppl 1):S35–41. 10865404

[63] Noppakun K Ingsathit A Pongskul C Premasthian N Avihingsanon Y Lumpaopong A and Subcommittee for Kidney Transplant Registry and Thai Transplantation Society. A 25-year experience of kidney transplantation in Thailand: report from the Thai Transplant Registry. Nephrology (2015) 20(3):177–83. 10.1111/nep.12378 25492162

[64] Tungsiripat R Tangcharoensathien V Regulation of Organ Transplantation in Thailand: Does it work? [HEFP Working Paper 04/03]. London: Health Economics and Financing Programme, London School of Hygiene and Tropical Medicine (2006). 33 p.

[65] Nivatvongs S Dhitavat V Jungsangasom A Attajarusit Y Sroyson S Prabjabok S Thirteen years of the Thai red cross organ donation centre. Transplant Proc (2008) 40(7):2091–4. 10.1016/j.transproceed.2008.06.032 18790161

[66] Kidney Foundation of Thailand. Kidney Foundation of Thailand, Thai Transplantaion Society and cooperated parties organizing “The Kidney Transplant Give a Royal Charity 60th Years of Her Royal Highness Princess Maha Chakri Sirindhorn” During April 2, 2015 – April 1, 2016 (in Thai). Bangkok (2016). Available from: http://www.kidneythai.org/newsdetail30.php

[67] Nivatvongs S Dhitavat V Jungsangasom A Attajarusit Y Sroyson S Prabjabok S Organ donation program to honor the 60th anniversary of the king’s accession to the throne. Transplant Proc (2008) 40(7):2095–6. 10.1016/j.transproceed.2008.06.012 18790162

[68] Thai Transplantation Society. 2015 Annual Report of Organ Transplantation in Thailand. Bangkok (2016). Available from: transplantthai.org/pic/File/2015%20Annual%20Report%20of%20Organ%20Transplantation%20in%20Thailand.pdf

[69] Thai Transplantation Society. 2014 Annual Report of Organ Transplantation in Thailand. Bangkok (2016). Available from: http://www.transplantthai.org/pic/File/Registry%202014(1).pdf

[70] Avihingsanon Y Townamchai N Nivatvongs S Ratchanon S Praditpornsilpa K Tiranathanagul K Living-donor kidney transplantation across ABO barriers: the first case in Thailand. Asian Biomed (Res Rev News) (2010) 3(5):525–9.

[71] The International Registry in Organ Donation and Transplantation. IRODaT Data as of October 4, 2016. Barcelona (2016). Available from: www.irodat.org

[72] Danguilan RA De Belen-Uriarte R Jorge SL Lesaca RJ Amarillo ML Ampil RS National survey of Filipinos on acceptance of incentivized organ donation. Transplant Proc (2012) 44(4):839–42. 10.1016/j.transproceed.2012.01.100 22564562

[73] De Villa V Alonzo H Tejada F Liquete R Purugganan B Alano F Characterization of kidney allograft donation in the Philippines. Transplant Proc (1997) 29(1–2):1584–5. 10.1016/S0041-1345(96)00683-5 9123433

[74] Naidas OD Chan-Licuanan KR Velasco VP Dalay CV Bayog DV Rosete-Liquete RM Cost effectiveness analysis of alternative treatments of end-stage renal disease: Philippine experience. Transplant Proc (1998) 30(7):3116 10.1016/S0041-1345(98)00956-7 9838375

[75] Republic of Philippines, Philippine Health Insurance Corporation. PHIC Circular No. 06, S. 2006: Clarifications on PhilHealth Benefits for Dialysis. Pasig City (2016). Available from: https://www.philhealth.gov.ph/circulars/2006/circ6_2006.pdf

[76] Philippines Health Insurance Cooperation. PhilHealth Extends Dialysis Coverage to 90 Days. Pasig City (2015). Available from: https://www.philhealth.gov.ph/news/2015/extends_dialysis.html

[77] National Kidney and Transplantation Institute. Historical Milestones. Quezon City (2016). Available from: http://www.nkti.gov.ph/about-us/historical-milestones

[78] National Kidney and Transplantation Institute. Kidney Transplant in a Globalizing World. Quezon City (2016). Available from: http://www.nkti.gov.ph/news/472-kidney-transplant-in-a-globalizing-world

[79] Republic of Philippines, Department of Health, Office of the Secretary. Establishment of a National Program for Sharing of Organs from Deceased Donors. Administrative Order. Manila (2010). Available from: http://hrlibrary.umn.edu/research/Philippines/DOH%20Adm.%20Order%202010-0019%20on%20Organ%20Trafficking.pdf

[80] de Castro LD. Organ donation in the Philippines: should the dead do more? Indian J Med Ethics (2014) 11(3):143–50. 25160964 10.20529/IJME.2014.039

[81] Republic of Philippines, House of representatives, Congress of the Philippines. Sixteenth Congress Second Regular Session. Manila (2015). Available from: http://www.congress.gov.ph/legisdocs/first_16/CR00596.pdf

[82] Manauis MN Pilar KA Lesaca R de Belen Uriarte R Danguilan R Ona E. A national program for nondirected kidney donation from living unrelated donors: the Philippine experience. Transplant Proc (2008) 40(7):2100–3. 10.1016/j.transproceed.2008.06.033 18790164

[83] Bagayaua G Organ Trade Continues Despite Ban on Transplantation to Foreigners. ANS-CBN News. Newsbreak (2009). Available from: http://news.abs-cbn.com/special-report/03/08/09/organ-trade-continues-despite-ban-transplantation-foreigners

[84] Padilla BS Regulated compensation for kidney donors in the Philippines. Curr Opin Organ Transplant (2009) 14(2):120–3. 10.1097/MOT.0b013e328329256f 19469027

[85] Republic of the Philippines, Department of Health, Office of the Secretary. Revised National Policy on Kidney Transplantation from Living Nonrelated Organ Donor and Its Implementing Structure. Administrative Order No.2008-0004. (2008). Available from: http://hrlibrary.umn.edu/research/Philippines/DOH%20Adm.%20Order%202010-0018%20on%20Organ%20Trafficking.pdf

[86] Conde CH Philippines Bans Kidney Transplants for Foreigners. The New York Times. Asia Pacific (2008). Available from: http://www.nytimes.com/2008/04/30/world/asia/30phils.html

[87] de Castro LD. The Declaration of Istanbul in the Philippines: success with foreigners but a continuing challenge for local transplant tourism. Med Health Care Philos (2013) 16(4):92932. 10.1007/s11019-013-9474-4 23423444

[88] Pamugas GE Arakama MH Danguilan RA Ledesma D Outcomes of kidney transplantations under the philippine health insurance corporation’s type Z benefit package at the national kidney and transplant institute, Philippines. Transplant Proc (2016) 48(3):852–4. 10.1016/j.transproceed.2015.12.095 27234751

[89] Pisani E Olivier Kok M Nugroho K Indonesia’s road to universal health coverage: a political journey. Health Policy Plan (2017) 32(2):267–76. 10.1093/heapol/czw120 28207049 PMC5400042

[90] The Economist Intelligence Unit. Universal Healthcare Coverage in Indonesia One Year On. (2014). Available from: http://pages.eiu.com/rs/eiu2/images/Universal%20healthcare%20coverage%20in%20Indonesia%E2%80%94One%20year%20on%20WEB.pdf

[91] Rokx C Schieber G Tandon A Harimurti P Somanathan A Health Financing in Indonesia: A Reform Road Map. Directions in Development; Human Development. Washington, DC: World Bank (2009). Available from: http://siteresources.worldbank.org/HEALTHNUTRITIONANDPOPULATION/Resources/Peer-Reviewed-Publications/HealthFinancinginIndonesiaAReformRoadMap2009.pdf

[92] Prodjosudjadi W Incidence, prevalence, treatment and cost of end-stage renal disease in Indonesia. Ethn Dis (2006) 16(2 Suppl 2):S2-14–6. 16774003

[93] Suhardjono. The development of a continuous ambulatory peritoneal dialysis program in Indonesia. Perit Dial Int (2008) 28(Suppl 3):S59–62. 18552266

[94] Markum HM Renal transplantation problem in Indonesia. Acta Med Indones (2004) 36(3):184–6. 15557691

[95] Nather A Yusof N Hilmy N Radiation in Tissue Banking: Basic Science and Clinical Applications of Irradiated Tissue Allografts. Singapore: World Scientific (2007). 64 p.

[96] Oxford Business Group. Indonesia’s Universal Health Care Goals. The Report: Indonesia 2015: Health. (2016). Available from: https://www.oxfordbusinessgroup.com/overview/indonesias-universal-health-care-goals

[97] Prodjosudjadi W Suhardjono A. End-stage renal disease in Indonesia: treatment development. Ethn Dis (2009) 19(1 Suppl 1):S1-33–6. 19484872

[98] World Bank Group. Viet Nam Overview. Washington, DC (2016). Available from: http://www.worldbank.org/en/country/vietnam/overview

[99] Ledinh H. Landmarks in clinical solid organ transplantation in Vietnam. Transplant Proc (2011) 43(9):3408–11. 10.1016/j.transproceed.2011.09.049 22099808

[100] Van Bui P. How peritoneal dialysis has developed in Vietnam. Perit Dial Int (2008) 28(Suppl 3):S63–6. 18552267

[101] Duong CM Olszyna DP Nguyen PD McLaws ML. Challenges of hemodialysis in Vietnam: experience from the first standardized district dialysis unit in Ho Chi Minh City. BMC Nephrol (2015) 16:122. 10.1186/s12882-015-0117-2 26231882 PMC4522093

[102] The National Assembly. Law on Donation, Removal and Transplantation of Human Tissues and Organs and Donation and Recovery of Cadavers. Pursuant to the 1992 Constitution of the Socialist Republic of Vietnam, Which Was Amended and Supplemented Under Resolution No. 51/2001/QH10 of December 25, 2001, of the Xth National Assembly, the 10th Session. Hanoi (2006). Available from: http://moj.gov.vn/vbpq/en/lists/vn%20bn%20php%20lut/view_detail.aspx?itemid=3780

[103] VUFO-NGO Resource Centre. Vietnam Sets up First Human Organ Transplant Center. Hanoi (2016). Available from: http://www.ngocentre.org.vn/news/vietnam-sets-first-human-organ-transplant-center

[104] Ledinh H Detry O Pham MS Truong HM Tran TP Nguyen PK Renal transplantation from living related donors: a single center experience in Viet Nam. Transplant Proc (2010) 42(10):4389–91. 10.1016/j.transproceed.2010.07.019 21168705

[105] Latt NN Myat Cho S Htun NM Yu MS Myint MN Aoki F Healthcare in Myanmar. Nagoya J Med Sci (2016) 78(2):123–34. 27303099 PMC4885812

[106] Saw YM Win KL Shiao LW Thandar MM Amiya RM Shibanuma A Taking stock of Myanmar’s progress toward the health-related Millennium Development Goals: current roadblocks, paths ahead. Int J Equity Health (2013) 12:78. 10.1186/1475-9276-12-78 24025845 PMC3847191

[107] Thin NN. An audit and comparative analysis of the kidney transplantation programme in Burma. Int J Surg (2004) 2(2):84–7. 10.1016/S1743-9191(06)60049-6 17462225

[108] Myint SYS Leading Doctors Call for Rethink on Transplant Laws. The Myanmar Times (2013). Available from: http://www.mmtimes.com/index.php/in-depth/86-main/6687-leading-doctors-call-for-rethink-on-transplant-laws.html

[109] Eleven Media Group. Myanmar Patients Prefer India for Organ Transplants. The Nation. Bangkok: Breakingnews, AEC (2013). Available from: http://www.nationmultimedia.com/news/breakingnews/aec/30197497

[110] World Health Organization, World Health Organization Regional Office for the Western Pacific. Report from the Consultation Meeting on Transplantation with National Health Authorities in the Western Pacific Region. Manila (2005). Available from: http://iris.wpro.who.int/handle/10665.1/6171

[111] Naramura T Hyodo T Kokubo K Matsubara H Wakai H Nakajima F Dialysis and quality of dialysate in southeast Asian developing countries. Nephron Extra (2014) 4(1):64–9. 10.1159/000362454 24926310 PMC4036132

[112] Working Group 8. Report of the Madrid Consultation: part 2: reports from the working groups. Transplantation (2011) 91(Suppl 11):S67–114. 10.1097/01.tp.0000399134.59371.56 21633284

[113] World Health Organization, Global Health Observatory Data Repository. Road Traffic Deaths, Data by Country. (2016). Available from: http://apps.who.int/gho/data/node.main.A997

[114] Suwanwela NC. Stroke epidemiology in Thailand. J Stroke (2014) 16(1):1–7. 10.5853/jos.2014.16.1.1 24741559 PMC3961816

[115] Bellingham JM Santhanakrishnan C Neidlinger N Wai P Kim J Niederhaus S Donation after cardiac death: a 29-year experience. Surgery (2011) 150(4):692–702. 10.1016/j.surg.2011.07.057 22000181 PMC3357118

[116] Steinbrook R Organ donation after cardiac death. N Engl J Med (2007) 357:209–13. 10.1056/NEJMp078066 17634455

[117] Morrissey PE Monaco AP. Donation after circulatory death: current practices, ongoing challenges, and potential improvements. Transplantation (2014) 97(3):258–64. 10.1097/01.TP.0000437178.48174.db 24492420

[118] Spital A Unrelated living donors: an update of attitudes and use among United States transplant centers. Transplantation (1994) 57:1722–6. 10.1097/00007890-199457120-00006 8016875

[119] Bia MJ Ramos EL Danovitch GM Gaston RS Harmon WE Leichtman AB Evaluation of living renal donors the current practice of US transplant centers. Transplantation (1995) 60(4):322–6. 10.1097/00007890-199508270-00003 7652758

[120] Jingwei AH Yu-Hung AL Ching L Living organ transplantation policy transition in Asia: towards adaptive policy changes. Glob Health Gov (2010) 3:1–14.22506090

[121] International Transplant Nurses Society. Position Statement of the International Transplant Nurses Society on Financial Incentives for Organ Donation. Chicago, IL (2015). Available from: http://www.itns.org/uploads/itns%20position%20statement%20final.pdf

[122] Matas AJ The rationale for incentives for living donors: an international perspective? Curr Transplant Rep (2015) 2:44 10.1007/s40472-014-0045-2

[123] Working Group on Incentives for Living Donation. Incentives for organ donation: proposed standards for an internationally acceptable system. Am J Transplant (2012) 12(2):306–12. 10.1111/j.1600-6143.2011.03881.x 22176925 PMC3350332

[124] Clarke KS Klarenbach S Vlaicu S Yang RC Garg AX Donor Nephrectomy Outcomes Research (DONOR) Network The direct and indirect economic costs incurred by living kidney donors – a systematic review. Nephrol Dial Transplant (2006) 21:1952–60. 10.1093/ndt/gfl069 16554329

[125] Warren PH Gifford KA Hong BA Merion RM Ojo AO. Development of the National Living Donor Assistance Center: reducing financial disincentives to living organ donation. Prog Transplant (2014) 24(1):76–81. 10.7182/pit2014593 24598569 PMC4374738

[126] Lau KK Butani L Increasing organ donation: we can do better! J Transplant Technol Res (2013) S2: e001 10.4172/2161-0991.S2-e001

[127] Delmonico FL Martin D Domínguez-Gil B Muller E Jha V Levin A Living and deceased organ donation should be financially neutral acts. Am J Transplant (2015) 15(5):1187–91. 10.1111/ajt.13232 25833381

[128] Sickand M Cuerden MS Klarenbach SW Ojo AO Parikh CR Boudville N Reimbursing live organ donors for incurred non-medical expenses: a global perspective on policies and programs. Am J Transplant (2009) 9(12):2825–36. 10.1111/j.1600-6143.2009.02829.x 19788503 PMC4388151

[129] Ladin K Wang R Hanto DW National healthcare policy, transplant-specific statutes, and the future of organ transplantation. In: Kirk AD Knechtle SJ Larsen CP Madsen JC Pearson TC Webber SA, editors. Textbook of Organ Transplantation. Oxford: John Wiley & Sons, Ltd (2014). p. 1709–15.

[130] Sharif MU Elsayed ME Stack AG. The global nephrology workforce: emerging threats and potential solutions! Clin Kidney J (2016) 9(1):11–22. 10.1093/ckj/sfv111 26798456 PMC4720191

[131] Loch A Hilmi IN Mazam Z Pillay Y Choon DSK Differences in attitudes towards cadaveric organ donation: observations in a multiracial Malaysia society. Hong Kong J Emerg Med (2010) 17(3):236–43.

[132] Wong LP Knowledge, attitudes, practices, and behaviors regarding deceased organ donation and transplantation in Malaysia’s multi-ethnic society: a baseline study. Clin Transplant (2011) 25:E22–31. 10.1111/j.1399-0012.2010.01312.x 20718827

